# Physical relaxation for occupational stress in healthcare workers: A systematic review and network meta‐analysis of randomized controlled trials

**DOI:** 10.1002/1348-9585.12243

**Published:** 2021-07-07

**Authors:** Michael Zhang, Brittany Murphy, Abegail Cabanilla, Christina Yidi

**Affiliations:** ^1^ Administration Division Southern Nevada Health District Las Vegas NV USA; ^2^ Department of Exercise Science Florida Atlantic University Boca Raton FL USA; ^3^ School of Life Sciences University of Nevada Las Vegas Las Vegas NV USA; ^4^ Department of Veterans Affairs Orlando VA Healthcare System Orlando FL USA

**Keywords:** burnout, healthcare workers, occupational medicine, stress

## Abstract

**Objectives:**

Work related stress is a major occupational health problem that is associated with adverse effects on physical and mental health. Healthcare workers are particularly vulnerable in the era of COVID‐19. Physical methods of stress relief such as yoga and massage therapy may reduce occupational stress. The objective of this systematic review and network meta‐analysis is to determine the effects of yoga, massage therapy, progressive muscle relaxation, and stretching on alleviating stress and improving physical and mental health in healthcare workers.

**Methods:**

Databases were searched for randomized controlled trials on the use of physical relaxation methods for occupational stress in healthcare workers with any duration of follow‐up. Meta‐analysis was performed for standard mean differences in stress measures from baseline between subjects undergoing relaxation vs non‐intervention controls. Network meta‐analysis was conducted to determine the best relaxation method.

**Results:**

Fifteen trials representing 688 healthcare workers were identified. Random‐effects meta‐analysis shows that physical relaxation methods overall reduced measures of occupational stress at the longest duration of follow‐up vs baseline compared to non‐intervention controls (SMD −0.53; 95% CI [−0.74 to −0.33]; *p* < .00001). On network meta‐analysis, only yoga alone (SMD −0.71; 95% CI [−1.01 to −0.41]) and massage therapy alone (SMD −0.43; 95% CI [−0.72 to −0.14]) were more effective than control, with yoga identified as the best method (*p*‐score = .89).

**Conclusion:**

Physical relaxation may help reduce occupational stress in healthcare workers. Yoga is particularly effective and offers the convenience of online delivery. Employers should consider implementing these methods into workplace wellness programs.

## 1 INTRODUCTION

Occupational stress has been recognized as one of the major occupational health problems affecting workers worldwide.^1^ Chronic exposure to work related stressors such as long hours and job strain has negative effects on physical and mental health.^2^, ^3^ Major causes of morbidity, including cardiovascular disease, diabetes, and depression, have been linked with work stress across multiple demographic groups,^3^, ^4^ and it has been estimated that employees with work stress suffer on average a 50% excess risk of coronary heart disease.^5^

Healthcare workers are an especially vulnerable group, with stressful environments and work pressure often leading to burnout.^6^, ^7^ Studies have assessed occupational stress in a wide range of workers, including nurses, physicians, technicians, therapists, and other personnel in various disciplines,^8^ and common themes have emerged in the literature. Long hours, overwork, shift work, inadequate staffing, emotional demands, administrative burdens, and physical workplace hazards are all believed to be contributors,^9^, ^10^, ^11^ and it has been suggested that stress and burnout have been associated with decreased job satisfaction, poor job performance, and negative patient outcomes.^12^, ^13^

The ongoing COVID‐19 pandemic has seen healthcare workers across the world brought under immense physical and emotion strain. From the early days of the COVID‐19 outbreak, surging case numbers placed increased demands on hospital staff and spawned a multitude of new challenges. Fear of infection, lack of adequate personal protective equipment (PPE), concerns for the health and safety of family and friends, limited training and experience against an emerging disease, and ever‐changing care protocols are several of the many sources of stress faced by frontline healthcare workers.^14^, ^15^ Numerous studies have been undertaken to assess the impact of these stressors; prevalence research from Japan,^16^ China,^17^ Italy,^18^ India,^19^ Iran,^20^ the United States,^21^ and other countries have documented high levels of anxiety, depression, stress, and burnout amongst healthcare workers, and a meta‐analysis using data from four continents found that the prevalence of each was in excess of 30%.^22^

Due to these concerns, a number of stress reduction techniques for healthcare workers involved in the COVID‐19 response have been recommended on both the individual and organization levels.^23^ Organizational approaches are aimed at improving work conditions and facilitating workflow,^24^ with particular emphasis placed on workplace safety and access to mental health.^23^ However, it is recommended that these be accompanied by strategies targeting the individual,^25^ which may also be incorporated into organizational stress reduction programs. These include mindfulness methods such as meditation, which promote awareness of the present moment without judgment so that arising stressors are met with calmness and equanimity,^26^ and cognitive behavioral approaches that “aim at changing cognitions and subsequently reinforcing active coping skills.”^27^

Another approach that may be helpful in alleviating work‐related stress is the use of physical methods such as yoga, massage therapy, and progressive muscle relaxation. Yoga has shown promise in a pilot crossover study in reducing occupational stress in Japanese nurses,^28^ and both massage and Pilates have been incorporated into a recently developed organizational program in France for hospital workers battling COVID‐19.^29^ These interventions fall under the umbrella of “physical relaxation” and were previously investigated in a 2015 Cochrane meta‐analysis which showed efficacy for stress reduction in healthcare workers compared to control at one month and one to six months follow‐up.^30^ However, this review included studies on music therapy,^31^ a quasi‐experimental study,^32^ and research involving an obscure auriculotherapy treatment,^33^ and combined post‐intervention and change scores as standardized mean differences, a practice that is no longer recommended.^34^ Furthermore, additional trials have appeared since this Cochrane review, and two recent systematic reviews have been qualitative and did not feature meta‐analyses.^35^, ^36^

Therefore, the objective of this study is to provide an updated systematic review and meta‐analysis of all randomized controlled trials of the use of physical methods of relaxation in healthcare workers on occupational stress reduction. We also examine the effect of relaxation methods on physical and mental health and compare various methods with each other and non‐intervention using network meta‐analysis (NMA).

## 2 METHODS

### 2.1 Search strategy and study selection

This meta‐analysis was conducted per the PRISMA (Preferred Reporting Items for Systematic Review and Meta‐analyses) guidelines.^37^ We sought to identify all randomized controlled trials of the use of physical relaxation methods compared to non‐intervention control or other physical relaxation methods for occupational stress in healthcare workers with change from baseline or both pre‐ and post‐intervention stress data at any duration of follow‐up, with the longest duration used for analysis. The intervention consists of physical relaxation, which is compared to non‐intervention or other physical relaxation controls. We defined physical relaxation as any method that involves light muscular tension and relaxation. This includes movement‐based techniques such as yoga and related exercises (eg tai chi and qigong), stretching, and walking, as well as passive techniques such as massage and progressive muscle relaxation. We excluded vigorous exercise, such as heavy aerobic activity and weightlifting. Techniques devoid of muscular activity, such as aromatherapy without massage and music therapy, were also excluded. The target population consisted of healthcare workers. We searched PubMed, SCOPUS, Web of Science, and the Cochrane Library from inception to February 21st, 2021 (date of search). The search strategy was as follows: (stress OR burnout) AND (healthcare OR healthcare worker) AND (yoga OR tai chi OR qigong OR massage OR exercise OR walk OR stretch OR muscle OR muscular OR relax OR therapy) AND trial under titles, abstracts, and keywords. Titles and abstracts were screened for eligibility, followed by full‐text assessment of potentially relevant articles. Finally, a manual search of references in pertinent review articles in this area was conducted for studies not found in the above databases. Studies were eligible for inclusion if they met all of the following criteria: full‐text English language articles published in peer reviewed journals, prospective RCT design with at least two arms; a physical relaxation intervention group and a non‐intervention control group or multiple physical relaxation groups (for the secondary analysis), study participants in both arms were all adult healthcare workers, at least one continuous measure of stress was reported with either changes from baseline reported in both arms or pre‐ and post‐intervention data available at any duration of follow‐up. The following were exclusion criteria: non‐RCT’s (such as quasi‐randomized and quasi‐experimental studies), lack of a non‐intervention or another physical relaxation comparison group, lack of stress assessment data or data that is otherwise insufficient for extraction, studies involving rigorous physical exercise or strength training, studies on subjects with preexisting mental illness, articles without full‐text, and non‐English manuscripts.

### 2.2 Primary and secondary outcomes

The primary outcome was the change in occupational stress after physical relaxation, and secondary outcomes were changes in physical and mental health. The follow‐up time frame of all outcome assessments was defined from the beginning of the intervention, for example, a study administering a follow‐up assessment for stress 2 weeks after the completion of 4 weeks of intervention is considered to have a 6‐week follow‐up. We conducted two types of meta‐analyses; pairwise two‐armed meta‐analyses which examined the effect of all methods of physical relaxation together on the primary and secondary outcomes, and a separate network meta‐analysis to compare individual modalities of physical relaxation with each other and non‐intervention control simultaneously on the primary outcome.

### 2.3 Data extraction

Two authors independently extracted data from all studies deemed eligible for inclusion, with disagreements addressed through discussion until a consensus was reached. The following data were obtained: nominal study characteristics including title, authors, journal, and year of publication; verification of RCT design, healthcare worker population under study, number of participants in each arm, type of intervention, type of stress assessment, and duration of follow‐up. If multiple follow‐up periods were reported, the longest was used for further analysis. For the primary outcome, we obtained mean changes of stress scores and standard deviation from baseline for both arms, with conversion from pre‐ and post‐intervention stress scores if not otherwise available using a correlation coefficient of zero.^38^, ^39^ When multiple scales are present in a study, preference was given to measures more specific for stress and those that are more commonly used in other identified studies. In decreasing order of preference, these are the Perceived Stress Scale (PSS), the Maslach Burnout Inventory for emotional exhaustion (MBI‐EE), the Nursing Stress Scale (NSS), and others if these are not available. Mean changes in assessments examining mental and physical health were obtained in similar fashion for the secondary outcome. Directional consistency of varying scales was ensured through multiplication of means by −1 where appropriate. Other measures of central tendency and variation were converted to means and standard deviation where appropriate per established methods.^39^, ^40^ Data from graphs were extracted with digitalization tools if not otherwise available.^41^

### 2.4 Quality assessment and risk of bias

Two authors independently assessed the quality of included RCT’s with the Cochrane Collaboration's Risk of Bias tool (RoB 2) per established recommendations.^42^ In brief, manuscripts were evaluated on five domains of bias: randomization process, deviations from intended interventions, missing outcome data, measurement of the outcome, and selection of the reported result. Domains were graded as “low risk,” “some concerns,” or “high risk,” with the overall bias determined by the highest grade in any domain. Disagreements were resolved through discussion until consensus was reached.

### 2.5 Statistical analysis

For the primary analysis, the pooled effects of physical interventions vs non‐intervention on the primary and secondary outcomes were analyzed with the meta‐package in RStudio version 1.4.1106. Data are reported as standardized mean differences (SMD) with 95% confidence intervals and presented on Forest plots. Random‐effects inverse variance models were used, and the *I* ^2^ test was used to assess study heterogeneity. Egger’s test was used to assess publication bias. Post hoc subgroup and meta‐regression analyses were performed to examine the influence of gender, control status, and duration of treatment on study heterogeneity. For the secondary analysis comparing various methods of physical relaxation to each other, a random‐effects frequentist network meta‐analysis was conducted with the R package netmeta and visualized with MetaInsight version 3.14.^43^ Sensitivity analysis was performed by temporarily omitting one study at a time to assess stability of results for both analyses. Statistical significance was set at *p* < .05.

## 3 RESULTS

### 3.1 Study selection

**FIGURE 1:**
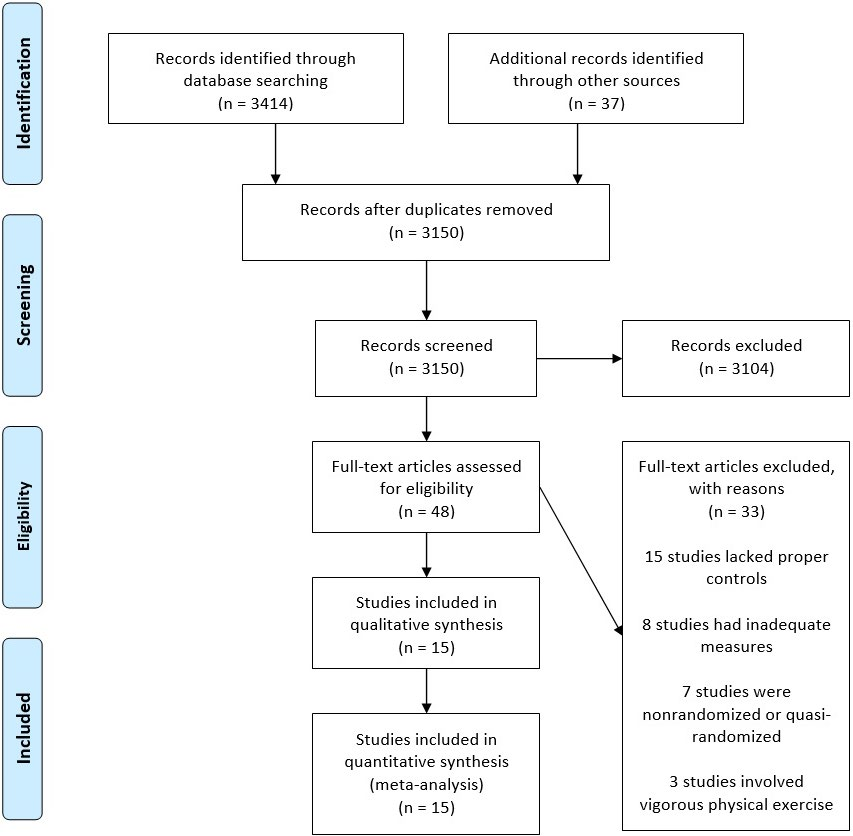
PRISMA study selection flowchart

We identified 3414 articles with the above search strategy, with another 37 through manual searching of review articles. After the removal of duplicates, 3150 records were screened. After the removal of 3102 nonrelevant records, 48 full text articles were assessed for eligibility. 33 of these records were filtered, with the most common reason being the lack of a non‐intervention control group or other physical relaxation comparison group (15 studies).^28^, ^44^, ^45^, ^46^, ^47^, ^48^, ^49^, ^50^, ^51^, ^52^, ^53^, ^54^, ^55^, ^56^, ^57^ Furthermore, seven studies were excluded due to non‐randomized or quasi‐randomized designs,^32^, ^58^, ^59^, ^60^, ^61^, ^62^, ^63^ eight for inadequate data,^64^, ^65^, ^66^, ^67^, ^68^, ^69^, ^70^, ^71^ and three due to interventions that included vigorous aerobic or weight training exercise.^72^, ^73^, ^74^ Finally, 15 studies that met our inclusion criteria were included in this meta‐analysis (Figure 1 and Table 1).^75^, ^76^, ^77^, ^78^, ^79^, ^80^, ^81^, ^82^, ^83^, ^84^, ^85^, ^86^, ^87^, ^88^, ^89^

**TABLE 1:** Characteristics of included studies

Study	Design	Population (*n* intervention/*n* control)	Control	Intervention	Outcome measures	Longest follow‐up after beginning treatment	Results
Bost et al. 2006^75^ (Australia)	RCT	Hospital nurses (27/21)	No therapy, controls asked to continue usual lifestyle	Swedish massage, 15 min weekly × 5 weeks	Trait‐STAI (State‐trait anxiety inventory)	5 weeks	Significant stress reduction in IG vs CG (*p* = .008)
Brennan et al. 2006^76^ (USA)	RCT	Hospital nurses (41/41)	No therapy, controls asked to take 10 min break	Chair massage for 10 min × 1	Perceived stress scale (PSS)	24 h	Stress reduction in IG vs CG at treatment end but no reduction from treatment end to 24 h follow‐up
Hansen et al. 2006^77^ (Norway)	RCT	Female psychiatric hospital nurses (18/14)	No therapy, controls promised treatment after study end	Aromatherapy massage, 90 min weekly × 6 weeks	Cooper’s job stress questionnaire (CSQ)	6 weeks	Significant stress reduction in IG (*p* = .007) but not in CG (*p* = .913)
Griffith et al. 2008^78^ (USA)	RCT	VA hospital staff (16/21)	Waiting list controls	Qigong, 60 min classes twice weekly plus 30 min self‐practice on non‐class days × 6 weeks	PSS, health status survey short form (SF‐36)	6 weeks	IG reduction in perceived stress vs CG (*p* = .02). No difference in mental or physical health
Palumbo et al. 2012^79^ (USA)	RCT	Female nurses in academic medical center aged ≥49 (6/5)	Controls promised a class after study end	Tai chi, 45 min weekly classes plus 10 min self‐practice at least 4 days a week × 15 weeks	PSS, SF‐36	15 weeks	No difference between IG and CG for stress, mental, and physical health (*p* = .42, 0.62, and 0.33 respectively).
Saganha et al. 2012^80^ (Portugal)	RCT	Physiotherapists suffering from burnout (8/8)	Waiting list controls	Qigong 20 min daily × 1 week followed by 5 min self‐practice twice daily × 2 more weeks	Maslach burnout inventory for emotional exhaustion (MBI‐EE)	3 weeks	Significant stress reduction in IG vs CG (*p* = .023)
Alexander et al. 2015^81^ (USA)	RCT	Hospital nurses (20/20)	Controls asked to continue usual self‐care	Yoga program × 8 weeks	MBI‐EE	8 weeks	Significant stress reduction in IG (*p* = .008) but not in CG
Lin et al. 2015^82^ (Taiwan)	RCT	Mental health professionals (30/30)	Controls watched television during tea break without exercise	Yoga, 60 min weekly classes × 12 weeks	Work‐related stress scale	12 weeks	Significant stress reduction in IG vs CG (*P* = .002)
Nazari et al. 2015^83^ (Iran)	RCT	ICU nurses (33/33)	No intervention	Swedish massage, 25 min twice weekly × 4 weeks	Occupational stress inventory (OSI)	6 weeks	Significant stress reduction in IG vs CG (*p* < .001)
Mathad et al. 2017^84^ (India)	RCT	Female nursing students (40/40)	Waiting list controls	Yoga, 60 min 5 days a week × 8 weeks	PSS	8 weeks	No significant stress reduction IG vs CG
Montibeler et al. 2018^85^ (Brazil)	RCT	Nurses and nursing technicians at surgical center (19/19)	Intervention made available to controls after study end	Aromatherapy massage, 10‐15 min × 6 sessions across 2 weeks	Work stress scale (WSS)	2 weeks	No difference in WSS after treatment
da Costa et al 2019^86^ (Brazil)	RCT	Nurses (20/19)	No intervention	Stretching, 40 min 3 times weekly × 8 weeks	Occupational stress scale (OSS)	8 weeks	Significant stress reduction in IG vs CG (*p* < .001)
Mahdizadeh et al. 2019^87^ (Iran)	RCT	Male EMS staff (29/29)	No intervention	Swedish massage, 20‐25 min twice weekly × 4 weeks	Expanded nurses’ occupational stress scale (ENSS)	4 weeks	Significant stress reduction in IG vs CG (*p* = .001)
Akyurek et al. 2020^88^ (Turkey)	RCT	Female hospital nurses (15/15)	Controls rested in reading room	Progressive muscle relaxation, breathing posture exercises, 40 min × 5 weeks	Visual analog scale (VAS)	52 weeks	Significant stress reduction in IG vs CG (*p* = .041)
Mandal et al. 2021^89^ (India)	RCT	Hospital nurses (19/32)	Waiting list controls	Yoga, 50 min twice weekly × 12 weeks	PSS	12 weeks	Significant stress reduction in IG vs CG (*p* < .0001)

Abbreviations: CG, control group; IG, intervention group.

### 3.2 Study characteristics

A total of 688 subjects were enrolled across these 15 studies, with 341 participants having undergone physical relaxation compared to 347 non‐intervention controls. Of the former group, 139 were involved in yoga or a yoga like exercise (tai chi and qigong); 167 received some type of massage therapy; 15 were engaged in progressive muscle relaxation (PMR), and 20 performed stretching exercises. All studies compared a physical relaxation intervention to non‐intervention. Follow‐up duration was one day to one year, with the remaining studies between 2 and 15 weeks. Of the studies reporting gender, the overwhelming majority (78.1%) of participants were female, and the average age was 30.8 years. Four studies specified waitlist controls, with another two that promised some form of intervention upon study completion. Five studies asked controls to take a break, relax, read, or go about their usual business, three studies did not specify control activities other than non‐intervention, and one study made interventions available at study end without explicitly promising beforehand. A wide variety of instruments were used for outcome measurements, with the most common being the Perceived Stress Scale (PSS) for stress assessment (five studies) followed by the Maslach Burnout Inventory for emotional exhaustion (MBI‐EE) (two studies). Physical and mental health were assessed through the Health Status Survey Short Form (SF‐36) in two studies. The most common healthcare profession represented was nursing; 11 studies with 575 participants consisted of nurses, nursing students, or nursing technicians.

### 3.3 Study quality

The risk of bias assessment is available in the supplementary file (Table S1). Most studies did not specify allocation methods, and all studies had performance bias due to self‐reporting of outcomes. Due to the nature of the interventions, providers and participants cannot be blinded. Overall, seven studies were judged to have high bias, eight with some concerns, and one with low bias.

### 3.4 Study outcomes

#### 3.4.1 Physical relaxation vs non‐intervention for occupational stress

**FIGURE 2:**
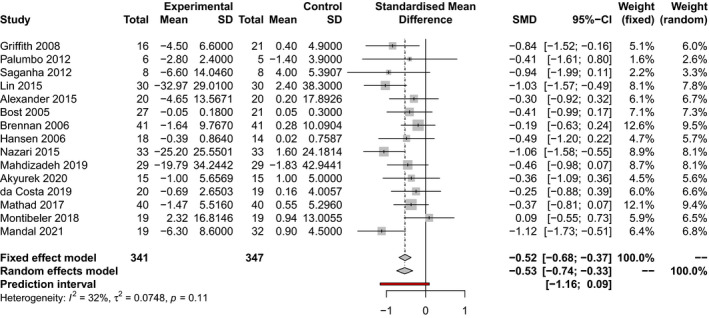
Meta‐analysis of all physical relaxation methods vs no intervention on occupational stress reduction at the longest duration of follow‐up from baseline. A negative SMD indicates a reduction in stress measures vs baseline

Random‐effects meta‐analysis of the 15 included trials totaling 688 healthcare workers is presented in (Figure 2). Pooled results show that altogether, interventions involving yoga (seven trials), massage therapy (six trials), PMR (one trial), and stretching exercises (one trial) significantly reduced measures of occupational stress at the longest duration of follow‐up vs baseline compared to non‐intervention controls (SMD −0.53; 95% CI [−0.74 to −0.33]; *p* < .00001). Moderate heterogeneity was observed across these studies (*I* ^2^ = 32%), and sensitivity analysis did not alter the results of the original analysis. Egger’s test did not suggest publication bias (*p* = .70).

#### 3.4.2 Subgroup and meta‐regression analyses

Due to the overwhelming female majority across these studies, a post‐hoc subgroup analysis was performed using the only two studies with appreciable numbers of males (25% or more) to examine the effect of gender. No significant difference was found between this subgroup, consisting of a study with 100% males^87^ and another with 42%,^83^ compared to the remaining nine studies with known gender compositions (92.4% female) (*p* = .31) (Figure S1). Four studies with indeterminant gender compositions were excluded from this analysis.^75^, ^80^, ^86^, ^89^ Another post‐hoc analysis was conducted to compare studies using waitlist controls with non‐waitlist control groups to address concerns that the former might overestimate intervention effects. A subgroup consisting of four studies explicitly utilizing waitlist controls combined with two additional studies promising therapy upon study conclusion compared with the nine remaining trials found no difference in treatment effect (*p* = .24) (Figure S2). Finally, mixed effects meta‐regression was used to investigate the contribution of treatment duration to between‐study differences in occupational stress measures at the longest duration of follow‐up. Despite the wide range of interventions from 10 min to 15 weeks, this was not found to be a significant source of inter‐trial heterogeneity (slope −0.044; 95% CI (−0.096 to −0.007); *p* = .086) (Figure S3). The results of these analyses are included in the supplementary file.

#### 3.4.3 Physical relaxation vs non‐intervention for physical and mental health

**FIGURE 3:**
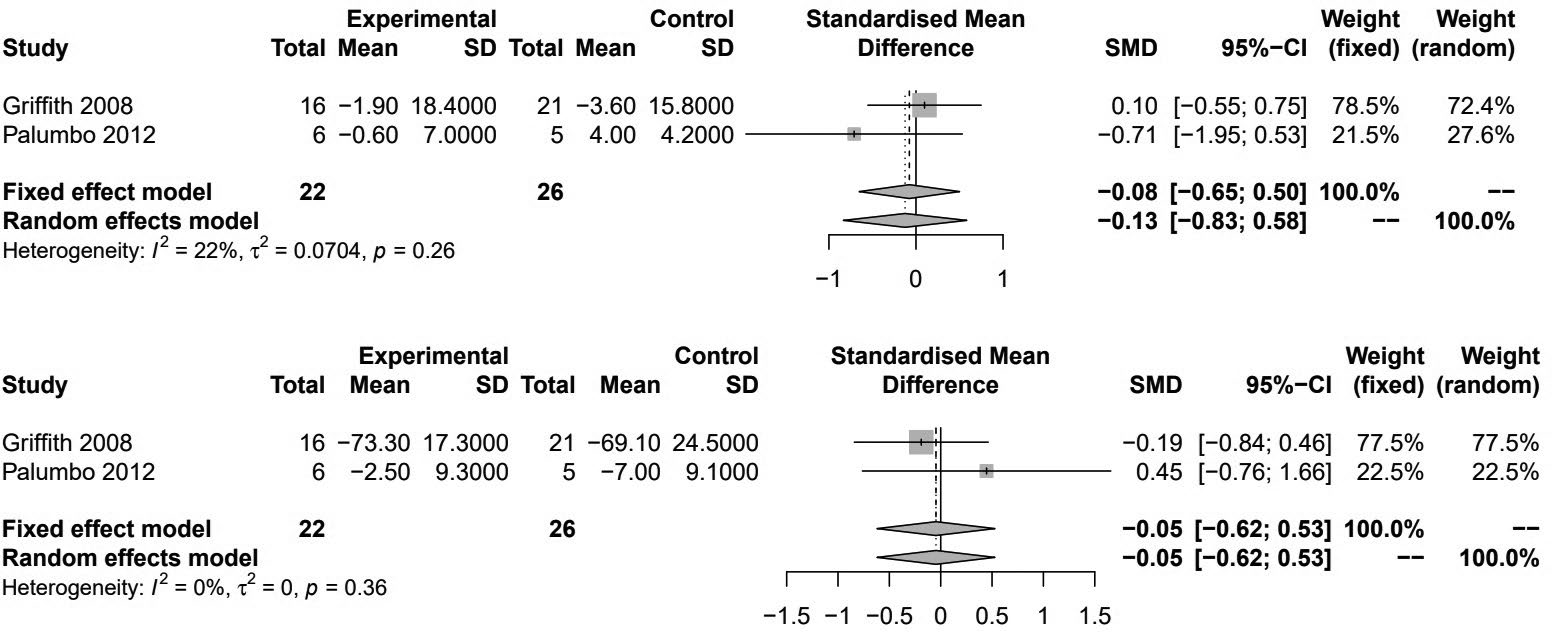
Meta‐analysis of physical relaxation methods vs no intervention on physical health (top) and mental health (bottom) at the longest duration of follow‐up from baseline

Figure 3 shows the results of meta‐analysis for the effects of physical relaxation on physical and mental health (two trials, 48 participants for both outcomes). Pooled SMD’s showed no difference between relaxation methods and no intervention for either outcome (SMD −0.13; 95% CI [−0.83 to 0.58]; *p* = .73) and (SMD −0.05; 95% CI [−0.62 to 0.53]; *p* = .87), respectively. These outcomes were measured by the SF‐36, which was multiplied by −1 to maintain consistent directionality. Study heterogeneity was low for both outcomes (*I* ^2^ = 22% and 0%, respectively), and no further analyses were conducted due to low study numbers.

#### 3.4.4 Network meta‐analysis of physical relaxation methods for occupational stress

**FIGURE 4:**
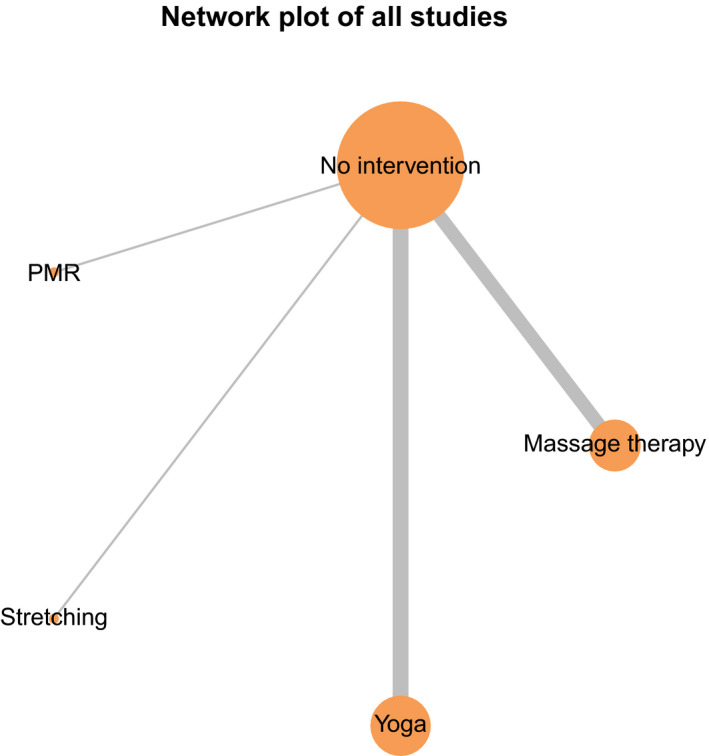
Network plot of physical relaxation trials. The size of each node is proportional to the sample size, and line thickness is proportional to the number of trials

The network plot of all 15 included trials is shown in (Figure 4). Although no studies directly compared various methods of physical relaxation with each other, all have been compared with the common comparator of non‐intervention, thus allowing for indirect between‐method comparisons. The size of the nodes are proportional to the number of participants in each intervention group, reflecting yoga (*n* = 139), massage (*n* = 167), stretching (*n* = 20), PMR (*n* = 15), and non‐intervention controls (*n* = 337). The number of trials studying each intervention type is reflected in the size of the lines connecting the nodes.

Random frequentist network meta‐analysis of these trials shows the relative effects of physical activity on occupational stress in ranked order (Table 2). Yoga was found to rank the highest in effectiveness, followed by massage therapy, PMR, stretching, and finally no intervention. Both yoga alone (SMD −0.71; 95% CI [−1.01 to −0.41]) and massage therapy alone (SMD −0.43; 95% CI [−0.72 to −0.14]) significantly reduced measures of occupational stress at the longest duration of follow‐up vs baseline compared to non‐intervention controls. The rank order of these interventions did not change on sensitivity analysis, and consistency was not evaluated due to the lack of direct comparisons.

**TABLE 2:** League table showing the results of network meta‐analysis comparing the effects of all methods of physical relaxation and control with SMD and 95% CI. Treatments are ranked from best to worst along the diagonal starting from the top left.

Yoga				
−0.28 [−0.70; 0.14]	Massage therapy			
−0.35 [−1.25; 0.56]	−0.06 [−0.96; 0.83]	PMR		
−0.46 [−1.29; 0.36]	−0.18 [−1.01; 0.64]	−0.12 [−1.27; 1.03]	Stretching	
−0.71 [−1.01; −0.41]*	−0.43 [−0.72; −0.14]*	−0.36 [−1.21; 0.48]	−0.25 [−1.02; 0.53]	No intervention

* Significant difference at the 95% confidence level.

Finally, we ranked individual interventions on the basis of their *p*‐score, which reflects the mean certainty that one treatment is better than other competing treatments, ranging continuously from 0 (least effective) to 1 (most effective).^90^ In agreement with the results of our network meta‐analysis, the ranked order of these interventions is yoga, massage therapy, PMR, stretching, and no‐intervention, with *p*‐scores of .89, .58, .51, .40, and .12 respectively.

## 4 DISCUSSION

Although numerous techniques of stress reduction in healthcare workers have been assessed in previous studies, physical methods of relaxation have produced consistently positive results. This meta‐analysis of multiple interventions confirms their overall effectiveness, with yoga and related exercises particularly beneficial. These findings are in agreement with systematic reviews in other professions and in the general population.^91^, ^92^, ^93^, ^94^

A notable feature of our study is that we have elected to conduct both pairwise and network meta‐analyses. The earlier Cochrane meta‐analysis utilized only the former, pooling results from both movement‐based and non‐movement‐based interventions.^30^ Sufficient homogeneity between interventions is a prerequisite for meaningful meta‐analysis,^34^ and this is reflected in various methods of physical relaxation. For example, yoga, which obviously incorporates stretching, has also been described as a form of “self‐massage”,^95^ and additionally has been compared to progressive muscle relaxation.^96^ Nevertheless, we detected moderate heterogeneity across trials. Possible causes explored were gender, control status, and treatment duration. Studies have suggested that the utilization of waitlist controls may overestimate treatment effects, as these participants appear to improve less than would otherwise be expected.^97^ However, we did not find a difference in effects between this subgroup and non‐waitlist controls. Similarly, there was no significant effect of gender and treatment duration found on subgroup and meta‐regression analyses. This leaves the possibility of variations in intervention modality,^98^ which would be identified on network meta‐analysis. We have ranked these interventions in order, with yoga and massage therapy superior to non‐intervention, yet we could not demonstrate that a given intervention was superior to another. The highest *p*‐score of yoga represents the highest probability of being the best treatment, but without information on ranking spread it cannot determine head‐to‐head superiority over another particular intervention.^90^, ^99^

The use of network meta‐analyses in the occupational medicine literature is apparently uncommon. The ability to assess multiple comparators simultaneously, rank them in order of effect, and elicit indirect evidence are powerful tools that may guide both interventional and preventive measures to promote worker health. Potential applications may include comparing treatments for work‐related injuries, return‐to‐work programs, and multiple workplace exposures. However, these techniques should not be used blindly. In addition to complex statistical assumptions that may necessitate expert input,^100^, ^101^ the nature of the primary research should be considered. A major assumption is the balanced distribution of effect modifiers such as study and patient characteristics across trials^102^; imbalance of these factors leads to bias and may invalidate study results.^103^ Interestingly, occupation itself can be considered as an effect modifier, and many studies use the occupational group as the unit of analysis,^104^ for example, assessing an intervention or exposure in nurses. Therefore, it may not be appropriate to compare such a study with one in a different occupation, even if the intervention or outcome were identical.

From the beginning of the COVID‐19 pandemic, there has been interest in developing both individual and organizational interventions that maintain the well‐being of hospital staff.^15^, ^23^, ^25^ There is debate over the relative efficacy of these approaches. Many reviews have emphasized individual therapy,^105^ but studies in the healthcare setting have noted that organizational interventions are longer lasting and more effective.^25^ However, this is not a mutually exclusive dichotomy. Workplace wellness programs are interventions implemented at the organizational level,^106^ but may incorporate relaxation methods targeting the individual. In France, these ideas were explored in the *Bulle* (bubble) program at Cochin Hospital in Paris, which supported hospital staff during the pandemic with a relaxing space that encouraged physical movement.^29^ Widespread adoption of such organizational programs should be encouraged by healthcare employers to promote employee wellness.

Stress reduction programs may be especially helpful for nurses. Nursing professionals comprise the great majority of subjects across these trials, and studies have consistently reported that nurses experience the highest levels of occupational stress and burnout of all healthcare workers.^107^, ^108^ Indeed, the profession has the distinction of having its own scales, the Nursing Stress Scale (NSS) and the expanded NSS (ENSS) for evaluating nursing occupational stress.^109^ Several job characteristics particular to the nursing profession may be contributing factors, such as work hours, time constraints, irregular schedules, and lack of professional support.^110^ Studies assessing nurses’ mental well‐being have found higher levels of anxiety,^111^ depression,^112^ and post‐traumatic stress^113^ compared to the general population. In our analyses, we were unable to compare nurses with non‐nursing professionals, as the only study that featured significant numbers of the latter consisted of physiotherapists with pre‐existing burnout.^80^

Our results suggest that yoga and related exercises may be the most effective methods of stress reduction. Several mechanisms have been postulated for these effects. Modulation of the autonomic nervous system appears to play a role, and studies have documented reductions in heart rate, blood pressure, and breath rate suggestive of reduced sympathetic and/or increased parasympathetic activity.^114^, ^115^ These peripheral effects are most likely mediated through vagal nerve stimulation,^116^ but central anxiolytic effects may also be produced through vagal communication with the nucleus tractus solitarii (NTS).^117^ Additional central effects include the release of beta‐endorphins and reduction in ACTH and cortisol levels.^118^, ^119^ Indeed, it has been found that yoga leads to significant reductions in salivary cortisol immediately after practice.^120^ Tai chi and qigong appear to operate via similar mechanisms.^121^ However, yoga stands out not only in terms of effectiveness but also in terms of the method of delivery. The recent need for social distancing has driven many activities online, and yoga enjoys obvious logistical advantages over massage therapy in keeping with these measures. Pilot studies of online yoga programs have shown improvements in mental well‐being in specialized populations,^122^, ^123^ and tele‐yoga has been suggested as a specific means of stress management in the era of COVID‐19.^124^

Our study has several limitations. The overall study quality of these trials was medium to low mainly due to lack of blinding and self‐reporting of measures which are largely unavoidable in this type of study. A recent review of 142 Cochrane meta‐analyses did not find a difference in treatment effect between trials with blinded and non‐blinded participants ^125^ and is thus unlikely to have influenced outcomes in this case. However, self‐reporting of outcomes has been associated with differing treatment effects,^126^, ^127^ and it is important for these outcome measures to be properly validated. Longitudinal validity is crucial in studies assessing pre‐ and post‐ outcomes,^128^ and the most commonly used scales in included trials have been validated in the healthcare‐worker population.^109^, ^129^, ^130^ In our study, an informal comparison between studies using the PSS, MBI, ENSS, and others did not find any subgroup differences (*p* = .99). There was only one trial identified each for PMR and stretching, and in the context of NMA, bias in only a single trial may affect multiple pooled estimates instead of just one in pairwise MA. Subjects were overwhelmingly young and healthy females who may not reflect the overall healthcare worker population. Although we performed a post hoc subgroup analysis to examine the effect of gender, the use of only two trials with appreciable numbers of males in this analysis makes it difficult to definitively conclude that these interventions are indeed gender neutral. As these two trials only evaluated massage therapy, it cannot be ruled out that other methods of relaxation may differ based on gender. We also did not find any benefit for these treatments on mental and physical health. One reason for this may be the low number of trials identified which studied these outcomes. Further, participants across all trials were generally young and healthy, and many trials specifically excluded subjects with any mental or severe physical illnesses. Identifying improvements in mental and physical health would be difficult in this context. For network meta‐analysis, we were unable to identify trials comparing multiple physical relaxation methods to each other instead of non‐intervention. Owing to the lack of direct evidence, we were unable to evaluate study consistency.

In some instances, multiple scales were used in the same trial. In such cases we used the scale that is more reflective of stress. For example, several studies used the MBI, which, in addition to the emotional exhaustion (MBI‐EE) component, also features the depersonalization (MBI‐DP) and personal accomplishment (MBI‐PA) scales. Since it has been suggested that MBI‐EE is a better measure of occupational stress,^131^ this scale only was chosen for meta‐analysis when data from all scales are available in the original trial. This is likewise true for the STAI state vs STAI trait; the latter may correlate more with the PSS^132^ and was thus the preferred scale. Occasionally, multiple measures were presented for occupational stress; in such situations, the most common scale was used; for example, in a trial featuring both the PSS and NSS,^79^ the PSS was used for meta‐analysis since it was used more frequently in other studies, perhaps leading to a trade‐off of consistency at the expense of specificity.

Finally, we have elected to group tai chi and qigong together with yoga under the common heading of yoga. Although these practices all possess their own distinct characteristics, from a physiological perspective they are hypothesized to act via similar mechanisms,^133^ and from a practical perspective each uses a combination of movement, breath, and “energy” to cultivate health benefits.^134^ Due to their similarities, other authors have applied phrases such as “meditative movement”^135^ and “contemplative activity”^133^ as umbrella terms, but we have selected yoga for simplicity. Regardless of nomenclature, it is clear that such methods offer unique benefits for stress reduction.

## 5 CONCLUSION

Healthcare workers face a multitude of stressors in their work environments. Occupational stress may lead to decreased job satisfaction, poor job performance, and impact overall health. Physical methods of relaxation may be helpful in reducing stress in this population. Movement‐based activities such as yoga are particularly effective and may be delivered remotely. Employers in the healthcare industry should consider implementing workplace wellness programs that integrate these methods to promote the well‐being of their staff.

## 6 ETHICS STATEMENT

Ethical approval is not required for this meta‐analysis as all data are collected and synthesized from previously published manuscripts.

## DISCLOSURES

Approval of the research protocol: N/A. Informed Consent: N/A. Registry and the Registration No. of the study/trial: N/A. Animal Studies: N/A.

## CONFLICT OF INTEREST

Authors declare no Conflict of Interests for this article.

## AUTHOR CONTRIBUTIONS

MZ contributed to study design, literature search, data collection, statistical analysis, and manuscript writing. BM contributed to study conceptualization, data collection, data interpretation, and manuscript editing. AC contributed to data interpretation, statistical analysis, and manuscript writing. CY contributed to study design, data collection, statistical analysis, and manuscript editing. All authors reviewed and approved the final manuscript.

### DATA AVAILABILITY STATEMENT

All data analyzed for this study are included in the manuscript.

## Supporting information

Supplementary Material
