# Supplementary material for: Physical relaxation for occupational stress in healthcare workers: A systematic review and network meta‐analysis of randomized controlled trials
Source: J Occup Health. 2021 Jul 7;63(1):e12243. doi: 10.1002/1348-9585.12243 (PMC8263904; doi:10.1002/1348-9585.12243)
Supplement: Supplementary file 1 — Supplementary Material [file JOH2-63-e12243-s001.docx]

| Author | Randomization process | Deviations from intended interventions | Missing outcome data | Measurement of the outcome | Selection of the reported result | Overall Bias |
| --- | --- | --- | --- | --- | --- | --- |
| Bost 2005^75^ | Low | Some concerns | High | Low | Some concerns | High |
| Brennan 2006^76^ | Some concerns | Some concerns | Low | High | Low | High |
| Hansen 2006^77^ | Some concerns | Some concerns | Low | Low | Low | Some concerns |
| Griffith 2008^78^ | Some concerns | Some concerns | Low | Low | Some concerns | Some concerns |
| Palumbo 2012^79^ | Some concerns | Some concerns | Some concerns | Low | Some concerns | Some concerns |
| Saganha 2012^80^ | Some concerns | Some concerns | Low | Low | Low | Some concerns |
| Alexander 2015^81^ | Some concerns | Some concerns | Low | Low | Low | Some concerns |
| Lin 2015^82^ | Low | Some concerns | Low | Low | Some concerns | Some concerns |
| Nazari 2015^83^ | Some concerns | Some concerns | High | Low | Low | High |
| Mathad 2017^84^ | Some concerns | Some concerns | High | low | Low | High |
| Montibeler 2018^85^ | Some concerns | Some concerns | Low | Low | Low | Some concerns |
| da Costa 2019^86^ | Some concerns | Some concerns | High | Low | Low | High |
| Mahdizadeh 2019^87^ | Some concerns | Some concerns | Low | Some concerns | Low | Some concerns |
| Akyurek 2020^88^ | Some concerns | Some concerns | High | Some concerns | Low | High |
| Mandal 2021^89^ | Low | Some concerns | High | Low | Some concerns | High |

Table S1. Risk of bias table

Figure S1. Forest plot of included studies with known gender composition assessing the effect of physical relaxation on occupational stress categorized by sex.

Figure S2. Forest plot of included studies assessing the effect of physical relaxation on occupational stress categorized by control status.

Figure S3. Meta-regression of included studies by weeks of intervention on stress outcomes at the longest duration of follow-up.
